# Supplementary material for: Single-cell TCR sequencing reveals phenotypically diverse clonally expanded cells harboring inducible HIV proviruses during ART
Source: Nat Commun. 2020 Aug 14;11:4089. doi: 10.1038/s41467-020-17898-8 (PMC7427996; doi:10.1038/s41467-020-17898-8)
Supplement: Supplementary file 1 — Supplementary Information [file 41467_2020_17898_MOESM1_ESM.pdf]

Supplementary files

Single-Cell TCR Sequencing Reveals Phenotypically Diverse Clonally Expanded Cells Harboring Inducible HIV Proviruses During ART

Gantner et al.

a

Well ID#	TRBV	CDR3 sequence (amino-acids)	TRBJ
A1	V6-5*01	CASRRAWRGALSNSPLHF	J1-6*01
A2	V6-5*01	CASRRAWRGALSNSPLHF	J1-6*01
A3	V4-1*01	CASSLTEAYGYTF	J1-2*01
A4	V20-1*01	CSAKDRVIETQFF	J2-5*01
A5	V29-1*01	CSVKDSYNEQFF	J2-1*01
A6	V6-5*01	CASRRAWRGALSNSPLHF	J1-6*01
A7	V10-3*01	CGVRDPFYPPHF	J1-5*01
A8	V6-5*01	CASRRAWRGALSNSPLHF	J1-6*01
A9	V6-5*01	CASRRAWRGALSNSPLHF	J1-6*01
A10	V6-5*01	CASRRAWRGALSNSPLHF	J1-6*01
A11	V6-5*01	CASRRAWRGALSNSPLHF	J1-6*01
A12	V4-1*01	CASSLTEAYGYTF	J1-2*01
B1	V6-5*01	CASRRAWRGALSNSPLHF	J1-6*01

Clonotype list:

- 1
- 2
- 3
- 4
- 5

b

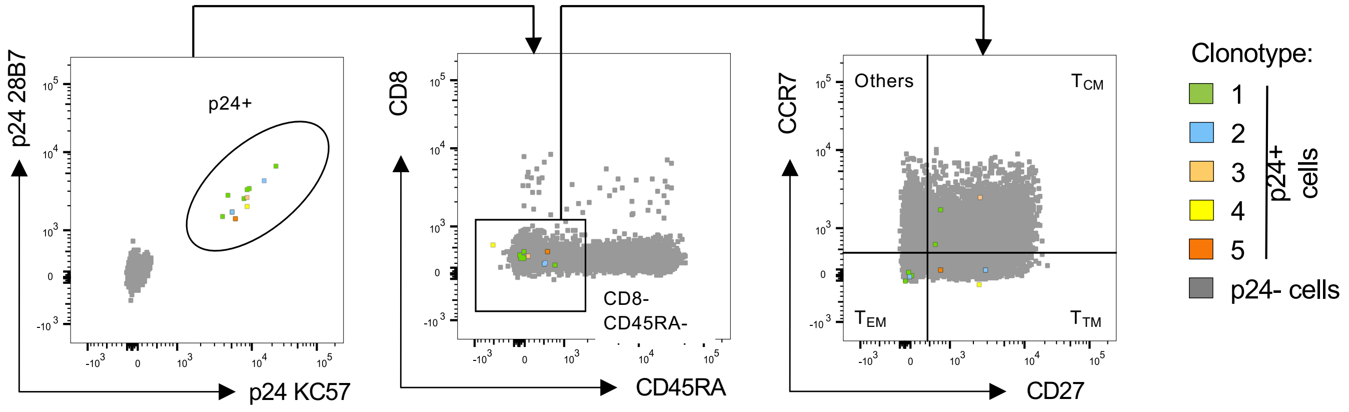

Supplementary Fig. 1. Phenotype and TCR sequences of individual p24+ cells from a representative participant. a. TRBV, TRBJ and CDR3 sequences from single sorted p24+ cells (n=13) from participant #1, visit 1. Sequences from two expanded clonotypes are shown in green and blue. **b.** Dot plots showing the level of expression of CD45RA, CCR7 and CD27 in individual p24+ cells (colored dots), overlaid onto total memory CD4+ T cells (in grey). The colors of individual p24+ cells correspond to the clonotypes shown in **a**.

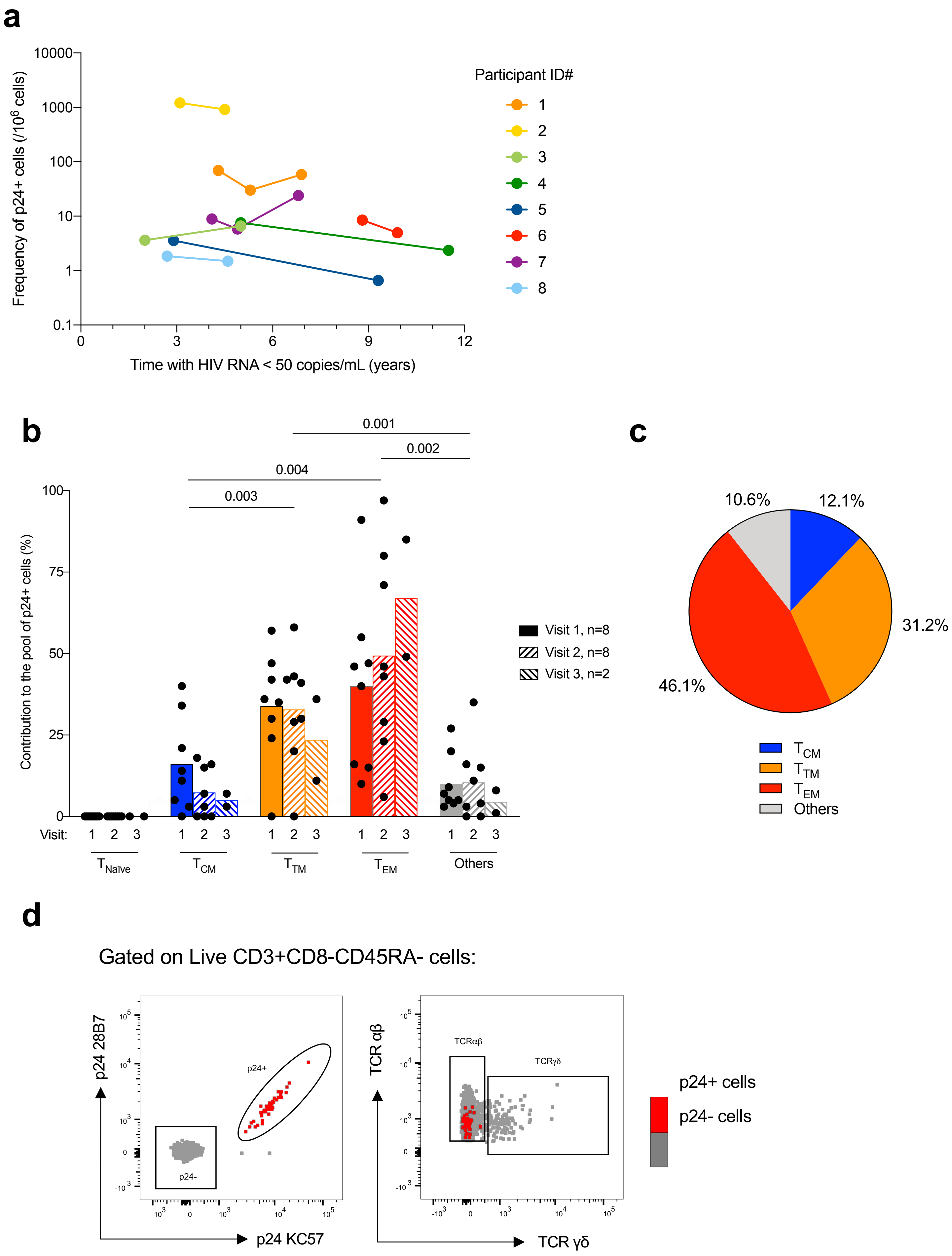
a
Frequency of p24+ cells (/10^6 cells)
10000
1000
100
10
1
0.1
0
3
6
9
12
Time with HIV RNA < 50 copies/mL (years)
Participant ID#
1
2
3
4
5
6
7
8
b
0.001
0.002
0.004
0.003
Contribution to the pool of p24+ cells (%)
100
75
50
25
0
Visit:
1 2 3
T Naïve
T CM
T TM
T EM
Others
Visit 1, n=8
Visit 2, n=8
Visit 3, n=2
c
10.6%
12.1%
31.2%
46.1%
T CM
T TM
T EM
Others
d
Gated on Live CD3+CD8-CD45RA- cells:
p24 28B7
p24+
p24-
p24 KC57
TCR αβ
TCRαβ
TCRγδ
TCR γδ
p24+ cells
p24- cells

Supplementary Fig. 2. Frequencies and phenotype of p24+ cells. a. Frequencies of p24+ cells in CD4+ T cells measured by HIV-Flow in longitudinal samples from all participants. **b.** Contribution of each memory subset to the pool of p24+ cells. Central memory (T_{CM}, blue); transitional memory (T_{TM}, orange); effector memory (T_{EM}, red); others (grey). Data are plotted for all study visits. The contribution to the pool of p24+ cells was higher for the most differentiated subsets (n=18 samples analyzed, two-sided Wilcoxon for paired comparisons). **c.** The mean contribution of each CD4+ T cell subset (T_{CM}, T_{TM}, T_{EM} and others) to the pool of p24+ cells is represented as a pie chart (all participants and all visits). **d.** Representative dot plots showing the expression of TCRαβ and TCRγδ in p24+ cells (in red), overlaid onto total memory CD4+ T cells (in grey).

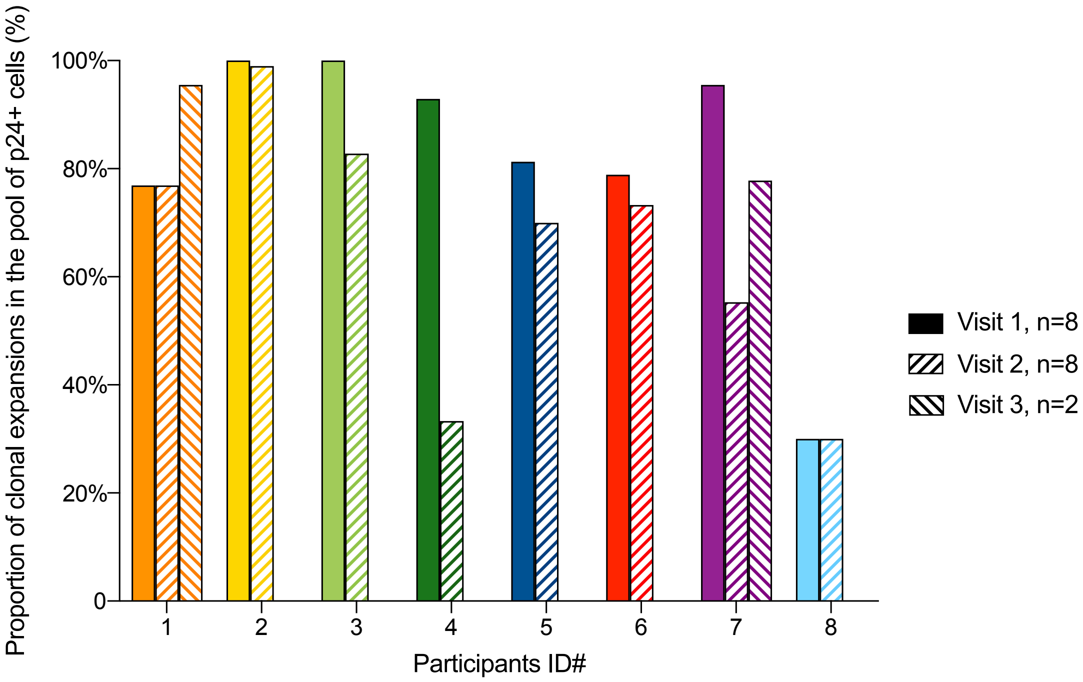

Supplementary Fig. 3. High proportion of clonal expansions in the translation-competent reservoir. Proportion of clonally expanded cells in the pool of p24+ cells for all participants and all study visits.

a

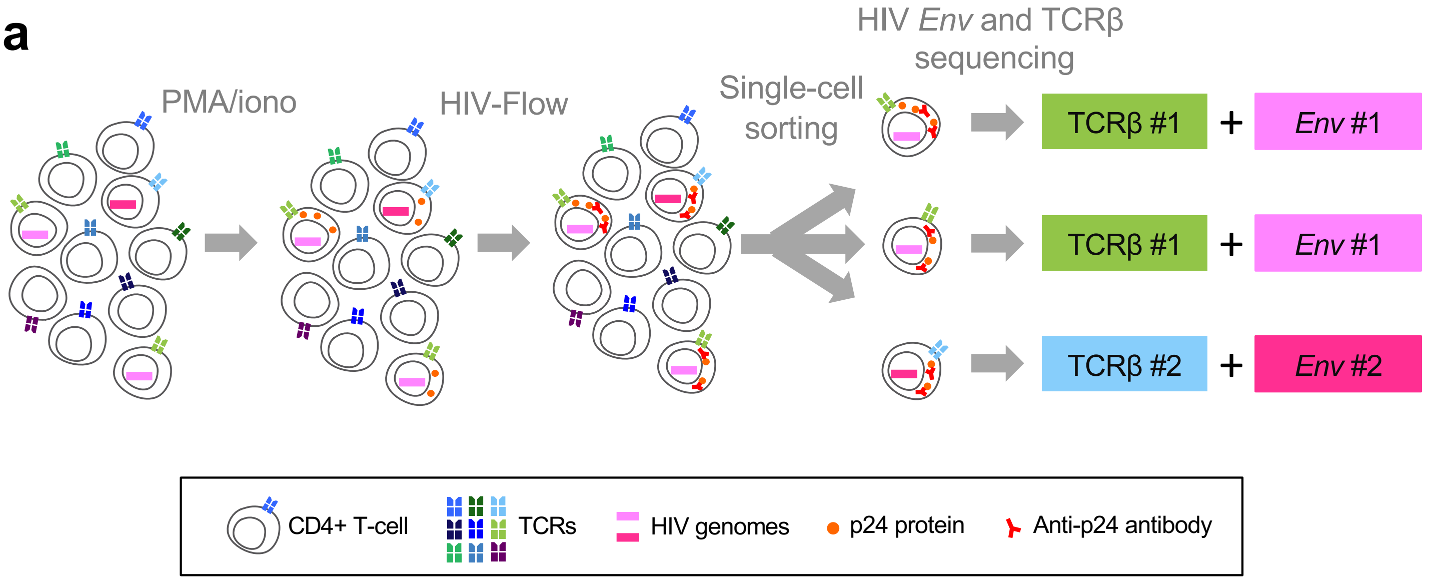

b

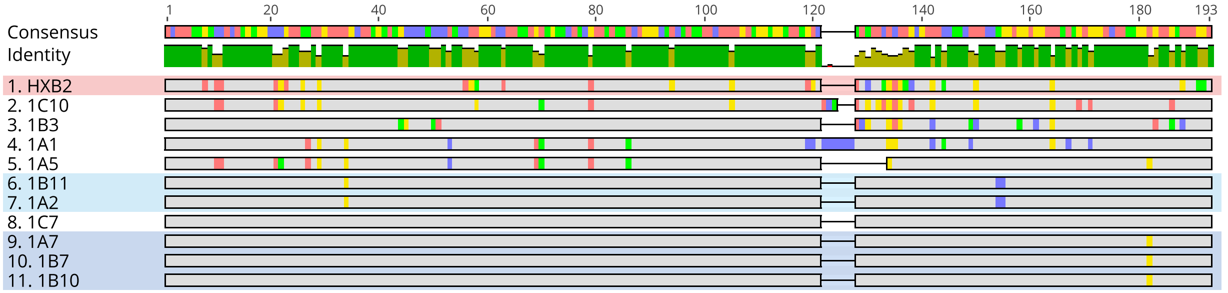

c

Env sequence

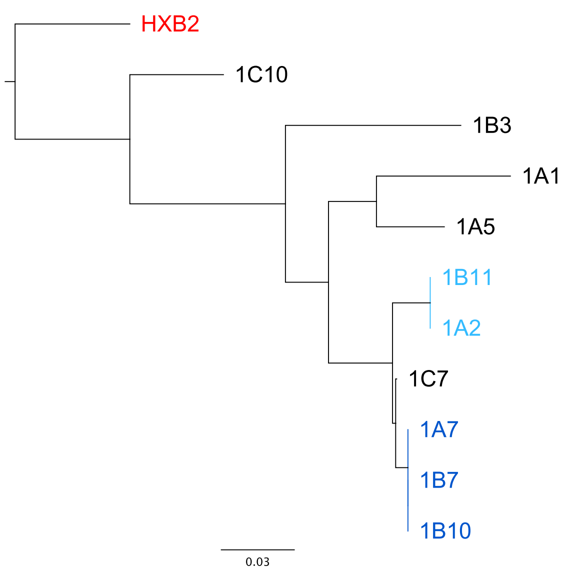

TCRβ sequence

Cell#	TRBV	CDR3	TRBJ
1C10	TRBV7-2	CASSFPAWTHFF	TRBJ2-5
1B3	TRBV6-2	CASSGTRBVRGNDQFF	TRBJ2-1
1A1	TRBV12-3	CASSTGTSRPTEQYF	TRBJ2-7
1A5	TRBV06-5	CASSTAGTRRPYYQYF	TRBJ2-4
1B11	TRBV30	CAWSTSGGPAGNEQFF	TRBJ2-1
1A2	TRBV30	CAWSTSGGPAGNEQFF	TRBJ2-1
1C7	TRBV11-3	CASSPSEQTYKEQFF	TRBJ2-1
1A7	TRBV18	CASSPSTTRBVKNEQFF	TRBJ2-1
1B7	TRBV18	CASSPSTTRBVKNEQFF	TRBJ2-1
1B10	TRBV18	CASSPSTTRBVKNEQFF	TRBJ2-1

Supplementary Fig. 4. Combined analyses of HIV *Env* and TCR sequences in single sorted p24+ cells. a. To obtain both TCR and HIV sequences from individual cells, we adapted our assay (described in Fig. 1a) to co-amplify TCRβ and HIV *Env* C3-V5 in a multiplex PCR. **b.** Alignment of *Env* sequences obtained from single-sorted p24+ cells (n=10). Two clusters of identical *Env* sequences (n=2 and n=3) were identified. All other sequences differed by at least 2 nucleotides. **c.** When pairing *Env* and TCR sequences, we observed that cells sharing the same *Env* sequence also shared the same TCR sequence (highlighted in blue).

Supplementary Fig. 5

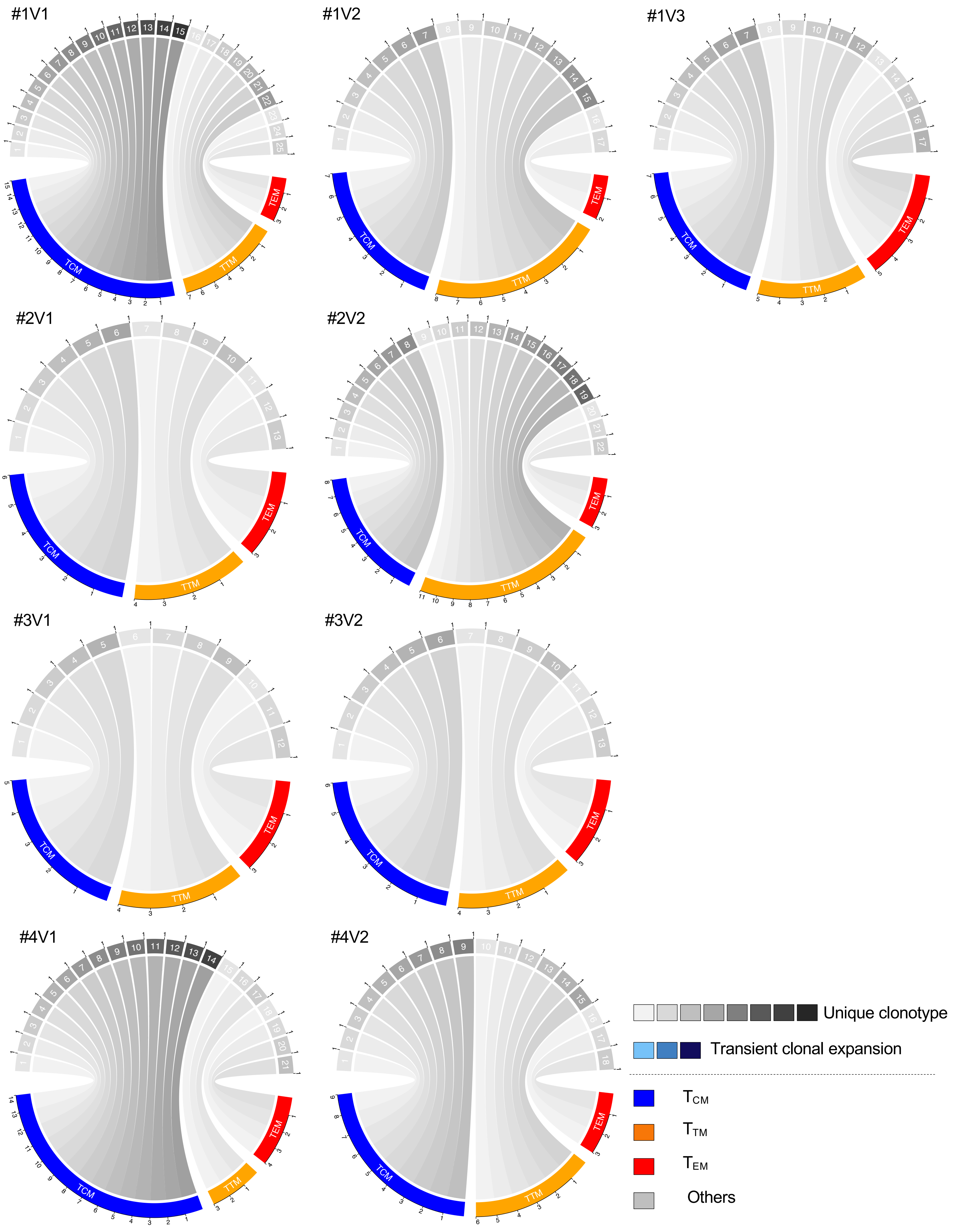

Supplementary Fig. 5 (cont.)

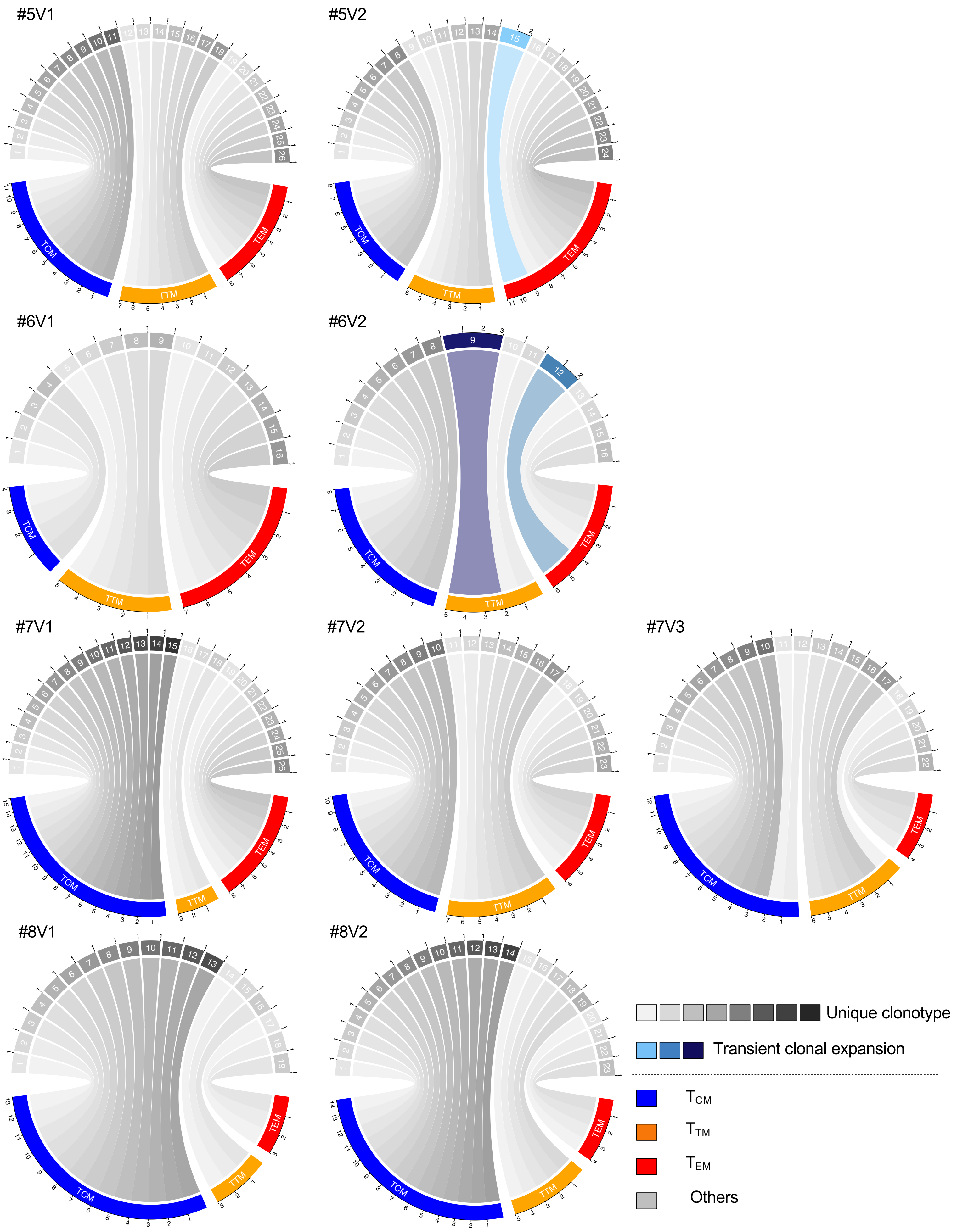

Supplementary Fig. 5. Phenotypic and clonotypic analysis of p24- cells. The distribution of TCRβ clonotypes of p24- cells among memory subsets is represented as a chord diagram for participants #1 to #8 and according to the study visit (V1, V2 and V3). The circular representation shows the link between a specific clonotype (top half of the circle) and its memory phenotype (bottom half of the circle). The circular axis represents the number of p24- cells in each clonotype/subset. Expanded clonotypes detected at a single visit are depicted in shades of blue; unique clonotypes are depicted in shades of grey. The memory subset color code is as follows: central memory (T_{CM}) in blue; transitional memory (T_{TM}) in orange; and effector memory (T_{EM}) in red.

Supplementary Fig. 6

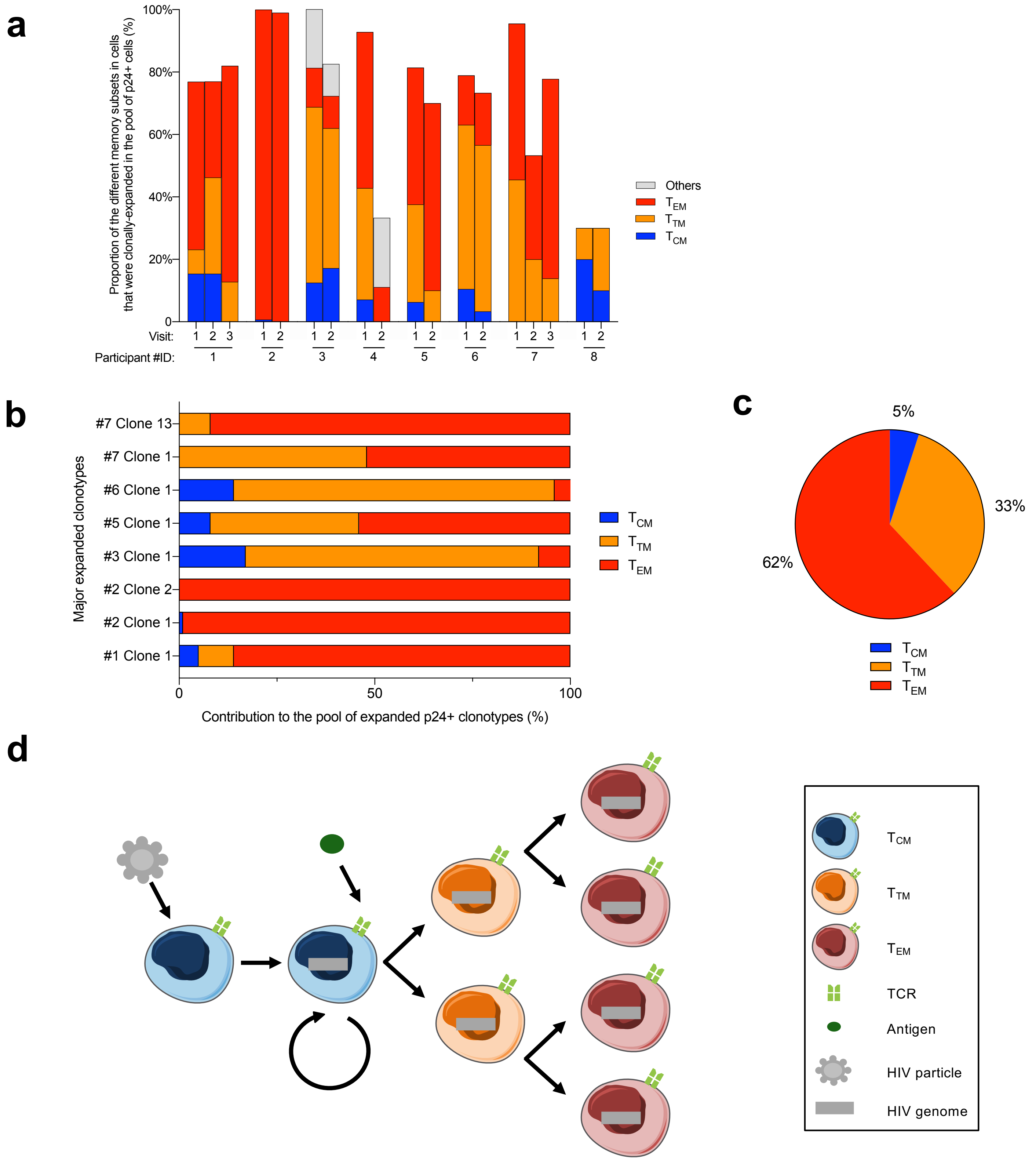

Supplementary Fig. 6. Major expanded p24+ clonotypes display multiple memory phenotypes. a. Proportion of the different memory phenotypes among cells that were clonally-expanded cells in the pool of p24+ cells for all participants and all study visits. **b.** Proportion of each memory subset in major clonally-expanded p24+ clonotypes (n≥10 p24+ cells). Major expanded infected clonotypes were systematically overrepresented in the most differentiated subsets (i.e. T_{TM} and T_{EM}) and displayed at least two different memory phenotypes. Rare T_{CM} cells were commonly observed within major expanded clonotypes. **c.** Pie chart depicting the mean contribution of each memory subset to the major expanded clones shown in **b**. **d.** The pool of HIV infected cells is maintained by cell proliferation. Whereas T_{TM} and T_{EM} cells represent the main contributors to the pool of infected cells, TCR analysis reveals that these cells are the progeny of infected T_{CM} cells that have long survival and self-renewal capacities. Our results suggest a model in which T_{CM} cells represent a long-lived source of reservoir cells that can expand upon antigen stimulation and replenish the pool of infected cells.

a

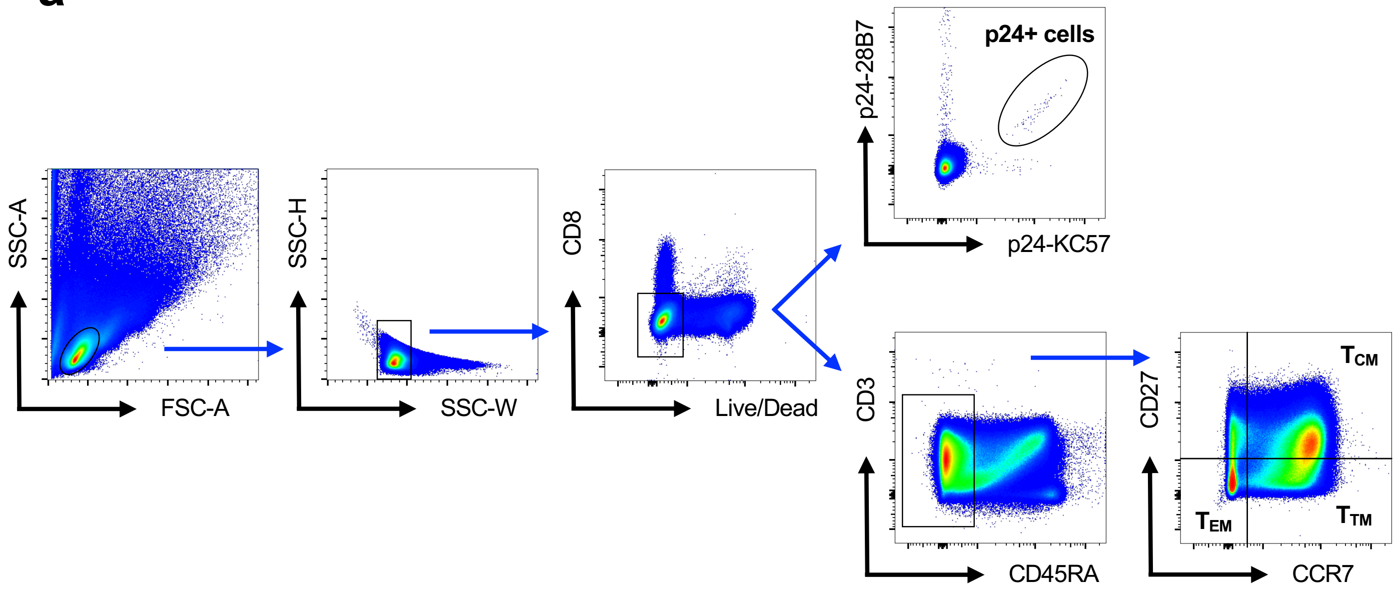

b

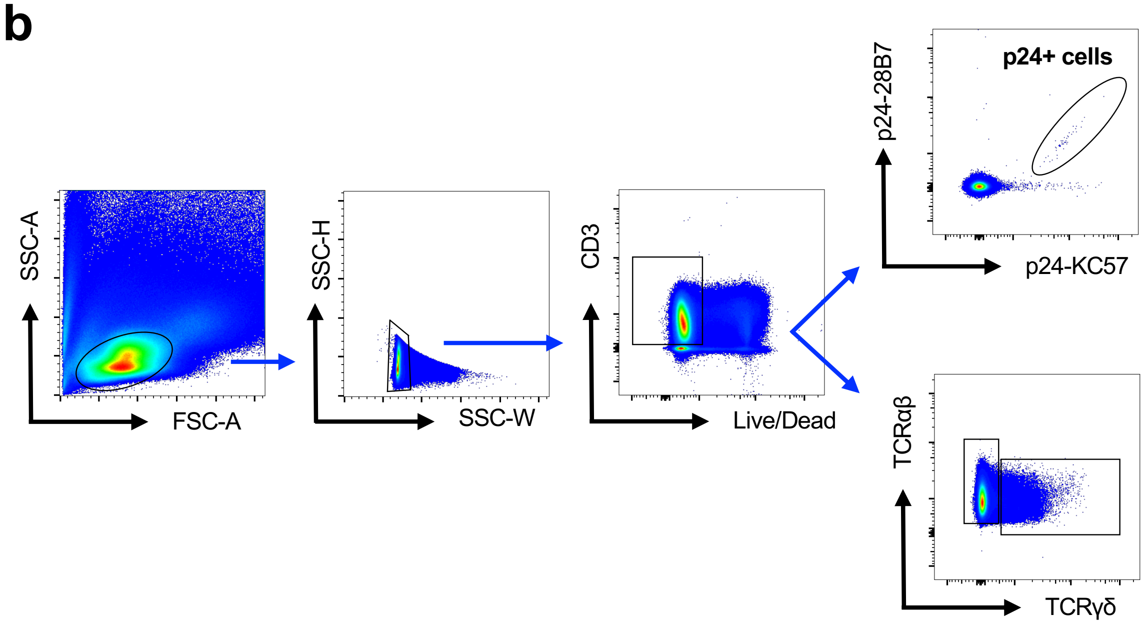

Supplementary Fig. 7. Gating strategies. a. Gating strategy used for index cell sorting of p24+ cells with recording of memory subsets. **b.** Gating strategy used to determine the TCR lineage of HIV p24+ cells.

Supplementary Table 1. Participants' characteristics

#ID	Sex	Ethnicity	HLA	Route of HIV acqui-sition	Nadir CD4 (cells/ mm^3)	Visit	Age (years)	CD4 (cells/ mm^3)	CD8 (cells/ mm^3)	HIV RNA (copies /mL)	Infection duration (years)	ART duration (years)	Undetectability duration (years)	Current ART	Total HIV DNA (cop./10^6 CD4 T cells)	Integrated HIV DNA (cop./10^6 CD4 T cells)	HIV-Flow (cells/10^6 CD4 T cells)
**1**	M	Hispanic	DRB1*01:01 DRB1*08:04	IVDU	384	1	57	587	945	<48	24.7	16.7	4.3	FPV/r+ABC+3TC	1601	1340	69.10
						2	58	612	834	<20	25.7	17.7	5.3	FPV/r+ABC+3TC	1311	1292	30.10
						3	60	677	788	<20	27.3	19.3	6.9	DRV/r+ABC/3TC	1249	1417	58.30
**2**	M	African American	DRB1*07:01 DRB1*13:04	HTS	21	1	39	267	518	<20	13.6	7.8	3.1	EFV/TDF/FTC	2749	3851	1208.00
						2	40	285	504	<20	15.0	9.2	4.5	EFV/TDF/FTC	3153	6059	914.60
**3**	M	Caucasian	DRB1*03:01 DRB1*08:02	MSM	186	1	43	1065	1408	<48	15.8	11.3	2.0	EFV/TDF/FTC	681	22	3.62
						2	46	887	1134	<20	18.8	14.3	5.0	EFV/TDF/FTC	640	12	6.61
**4**	M	Caucasian	DRB1*10:01 DRB1*13:02	MSM	70	1	48	268	673	<20	23.0	5.3	5.0	RAL+ETR+MVC	427	310	7.58
						2	55	316	733	<20	29.5	11.8	11.5	RAL+ETR+MVC	596	444	2.36
**5**	M	African American	DRB1*13:01 DRB1*15:03	MSM	181	1	32	248	378	<20	3.7	3.2	2.9	EFV/TDF/FTC	1945	1267	3.58
						2	39	420	568	<20	10.0	9.5	9.3	EVG/c/TAF/FTC	921	639	0.66
**6**	M	Caucasian	DRB1*04:04 DRB1*15:01	MSM	386	1	66	601	819	<40	22.2	15.7	8.8	DRV/r	2704	1424	8.49
						2	67	602	880	<40	23.3	16.8	9.9	DRV/r	2142	658	4.97
**7**	M	Caucasian	DRB1*01:02 DRB1*15:01	HTS	377	1	36	471	322	<40	10.5	4.2	4.1	RPV/TDF/FTC	221	175	8.87
						2	37	377	222	<40	11.3	5.1	4.9	RPV/TDF/FTC	262	229	5.83
						3	39	445	227	<40	13.2	7.0	6.8	RPV/TDF/FTC	354	106	23.90
**8**	F	African American	DRB1*03:02 DRB1*09:01	HTS	29	1	44	460	1879	<20	5.5	5.5	2.7	FPV/r+RAL+FTC/TDF	3300	2937	1.85
						2	46	541	1729	<20	7.4	7.4	4.6	DTG+TDF/FTC	3300	3239	1.49

M, male; F, female; IVDU, intravenous drug user; MSM, men who have sex with men; HTS, heterosexual; cop., copies; Und., Undetectability; ART, antiretroviral therapy; ABC, abacavir; FPV, fosamprenavir; DRV, darunavir; DTG, dolutegravir; EFV, efavirenz; ETR, etravirine; EVG, elvitegravir; FTC, emtricitabine; MVC, maraviroc; /r, ritonavir; RAL, raltegravir; RPV, rilpivirine; TAF, tenofovir alafenamide; TDF, tenofovir disoproxil fumarate; 3TC, lamivudine.

Supplementary Table 2. Primers used for amplification and sequencing of TCRβ

Name	Sequence 5'-3'
**PCR1 : Forward primers (tagged with M13F)**	
**VB2**	GTAAAACGACGGCCAGTATACTTCTATTGGTACAGACAAATCTTGG
**VB3**	GTAAAACGACGGCCAGTCTATGTATTGGTATAAACAGGACTCTAAG
**VB4**	GTAAAACGACGGCCAGTCAYARSGCTATGTATTGGTACAAGC
**VB5/9**	GTAAAACGACGGCCAGTCACTGTGTCCTGGTACCAACAG
**VB6**	GTAAAACGACGGCCAGTTACATGTACTGGTATCGACAAGACC
**VB7**	GTAAAACGACGGCCAGTTACCCTTTATTGGTACCGACAGAGCCTGG
**VB11**	GTAAAACGACGGCCAGTCTTTACTGGTACCGGCAGAWCYTGG
**VB12**	GTAAAACGACGGCCAGTTTTTCTGGTACAGACAGACCATGATG
**VB13**	GTAAAACGACGGCCAGTCACTGTCTACTGGTACCAGCAGG
**VB14**	GTAAAACGACGGCCAGTTGGACATGATAATCTTTATTGGTATCGAC
**VB15**	GTAAAACGACGGCCAGTCATGTACTGGTACCAGCAGAAGTC
**VB16**	GTAAAACGACGGCCAGTGTTATGTTTTTTGGTACCAACAGGTCC
**VB17**	GTAAAACGACGGCCAGTCATGTTTGTTCACTGGTACCGACAGAATC
**VB18**	GTAAAACGACGGCCAGTAGTCATGTTTACTGGTATCGGCAGC
**VB19**	GTAAAACGACGGCCAGTTGCCATGTACTGGTACCGACAG
**VB20**	GTAAAACGACGGCCAGTCACAACTATGTTTTGGTATCGTCAG
**VB21**	GTAAAACGACGGCCAGTTAGTTATGTTTACTGGTATCATAAGACGC
**VB23**	GTAAAACGACGGCCAGTATACTTTTTGTTTATTGGTATCAACAGAATCAG
**VB24**	GTAAAACGACGGCCAGTATGTACTGGTATCGACAAGACCC
**VB25**	GTAAAACGACGGCCAGTTGACAAAATGTACTGGTATCAACAAGATC
**VB29**	GTAAAACGACGGCCAGTTGATGTTCTGGTACCGTCAGCAAC
**VB30**	GTAAAACGACGGCCAGTCAACCTATACTGGTACCGACAGG
**PCR1 : Reverse primers (tagged with M13R)**	
**JB1-1**	CAGGAAACAGCTATGACCAACTGTGAGTCTGGTGCCTTGTCCAAAG
**JB1-2**	CAGGAAACAGCTATGACAACCTGGTCCCCGAACCGAAGG
**JB1-3**	CAGGAAACAGCTATGACAACAGTGAGCCAACTTCCCTCTCCAAAATA
**JB1-4**	CAGGAAACAGCTATGACCAGAGAGCTGGGTTCCACTGCCAAAAAACA
**JB1-5**	CAGGAAACAGCTATGACAGAGTCGAGTCCCATCACCAAAATGC
**JB1-6**	CAGGAAACAGCTATGACCTGGTCCCATTCCCAAAGTGGAGG
**JB2-1**	CAGGAAACAGCTATGACAGCCGTGTCCCTGGCCCGAAGAAC
**JB2-2**	CAGGAAACAGCTATGACCGTTTTTGGAGAAGGCTCTAGGCTGACC
**JB2-3**	CAGGAAACAGCTATGACCAGCCGGGTGCCTGAGCCAAAATAC
**JB2-4**	CAGGAAACAGCTATGACCGGGTCACGGCGCCGAAGTAC
**JB2-5**	CAGGAAACAGCTATGACAGCCGCGTCCTGGCCCGAAG
**JB2-6**	CAGGAAACAGCTATGACCTGCCGGCCCCGAAAGTCAGG
**JB2-7**	CAGGAAACAGCTATGACCCTGGTGCCCGACCCGAAG
**PCR2 and Sequencing: Forward and Reverse primers**	
**M13F**	GTAAAACGACGGCCAGT
**M13R**	CAGGAAACAGCTATGAC
**PCR1 : HIV Env C3-V5**	
**OutC3F**	CCTCAGGAGGGGACCCAGAA
**OutV5R**	TGGGTGCTACTCCTAATGGTTCA
**PCR2 and Sequencing : HIV Env C3-V5**	
**InC3F**	AGGGGAATTTTTCTACTGTAA
**InV5R**	ACTTCTCCAATTGTCCCTCA
