# Supplementary material for: Single-cell TCR sequencing reveals phenotypically diverse clonally expanded cells harboring inducible HIV proviruses during ART
Source: Nat Commun. 2020 Aug 14;11:4089. doi: 10.1038/s41467-020-17898-8 (PMC7427996; doi:10.1038/s41467-020-17898-8)
Supplement: Supplementary file 3 — Reporting Summary [file 41467_2020_17898_MOESM3_ESM.pdf]

nature research

Corresponding author(s): *Double-blind peer review submissions: write DBPR and your manuscript number here instead of author names.*

Last updated by author(s): Jun 5, 2020

Reporting Summary

Nature Research wishes to improve the reproducibility of the work that we publish. This form provides structure for consistency and transparency in reporting. For further information on Nature Research policies, see our Editorial Policies and the Editorial Policy Checklist.

Statistics

For all statistical analyses, confirm that the following items are present in the figure legend, table legend, main text, or Methods section.

n/a	Confirmed	
☐	☒	The exact sample size (*n*) for each experimental group/condition, given as a discrete number and unit of measurement
☐	☒	A statement on whether measurements were taken from distinct samples or whether the same sample was measured repeatedly
☐	☒	The statistical test(s) used AND whether they are one- or two-sided<br>*Only common tests should be described solely by name; describe more complex techniques in the Methods section.*
☐	☒	A description of all covariates tested
☒	☐	A description of any assumptions or corrections, such as tests of normality and adjustment for multiple comparisons
☐	☒	A full description of the statistical parameters including central tendency (e.g. means) or other basic estimates (e.g. regression coefficient) AND variation (e.g. standard deviation) or associated estimates of uncertainty (e.g. confidence intervals)
☒	☐	For null hypothesis testing, the test statistic (e.g. *F*, *t*, *r*) with confidence intervals, effect sizes, degrees of freedom and *P* value noted<br>*Give P values as exact values whenever suitable.*
☒	☐	For Bayesian analysis, information on the choice of priors and Markov chain Monte Carlo settings
☒	☐	For hierarchical and complex designs, identification of the appropriate level for tests and full reporting of outcomes
☒	☐	Estimates of effect sizes (e.g. Cohen's *d*, Pearson's *r*), indicating how they were calculated

Our web collection on statistics for biologists contains articles on many of the points above.

Software and code

Policy information about availability of computer code

Data collection	BD FACSDiva software
Data analysis	R version 3.1.1 (R Foundation, Vienna, Austria); Circlize package (version 0.4.8); Alluvial package (version 0.1-2); FlowJo v10.5.3; Graphpad Prism v6.0h.

For manuscripts utilizing custom algorithms or software that are central to the research but not yet described in published literature, software must be made available to editors and reviewers. We strongly encourage code deposition in a community repository (e.g. GitHub). See the Nature Research guidelines for submitting code & software for further information.

Data

Policy information about availability of data

All manuscripts must include a data availability statement. This statement should provide the following information, where applicable:

- Accession codes, unique identifiers, or web links for publicly available datasets
- A list of figures that have associated raw data
- A description of any restrictions on data availability

All data generated or analyzed during this study are included in this published article (and its supplementary information files). Source data are provided with this paper within Supplementary Data 1 and 2.
External databases used in this study are available online: IMGT® database (IMGT®, the international ImMunoGeneTics information system® [http://www.imgt.org]); McPAS-TCR database ([http://friedmanlab.weizmann.ac.il/McPAS-TCR/]).

Field-specific reporting

Please select the one below that is the best fit for your research. If you are not sure, read the appropriate sections before making your selection.

- [x] Life sciences
- [ ] Behavioural & social sciences
- [ ] Ecological, evolutionary & environmental sciences

For a reference copy of the document with all sections, see nature.com/documents/nr-reporting-summary-flat.pdf

Life sciences study design

All studies must disclose on these points even when the disclosure is negative.

Sample size	This is a descriptive study on 8 participants living with HIV on succesfull antiretroviral therapy and from whom multiple leukaphereses were obtained longitudinally. Samples from only eight patients were analyzed due to cost and the rarity of longitudinal samples available. No sample- size calculation was performed.
Data exclusions	No data were excluded from the analysis.
Replication	We repeated one sample (HIV-Flow combined with single TCR sequencing) in three independent experiments. We observed a similar distribution of the T cell clones within the reservoir, and we believe this demonstrates the reproducibility of our approach.
Randomization	Participants all belong to the same experimental group.
Blinding	This study is not randomized (see above), so no blinding is needed

Reporting for specific materials, systems and methods

We require information from authors about some types of materials, experimental systems and methods used in many studies. Here, indicate whether each material, system or method listed is relevant to your study. If you are not sure if a list item applies to your research, read the appropriate section before selecting a response.

Materials & experimental systems

n/a	Involved in the study	
[ ]	[x]	Antibodies
[x]	[ ]	Eukaryotic cell lines
[x]	[ ]	Palaeontology and archaeology
[x]	[ ]	Animals and other organisms
[ ]	[x]	Human research participants
[x]	[ ]	Clinical data
[x]	[ ]	Dual use research of concern

Methods

n/a	Involved in the study	
[x]	[ ]	ChIP-seq
[ ]	[x]	Flow cytometry
[x]	[ ]	MRI-based neuroimaging

Antibodies

Antibodies used	p24 KC57-PE : Beckman Coulter (clone: KC57; cat# 6604667)<br>p24 28B7-APC : MediMabs (clone: 28B7; cat# MM-0289-APC)<br>CD3-AF700 : BD Bioscience (clone: UCHT-1; cat#557943)<br>CD8-FITC : Miltenyi/MACS (clone: BW135/80; cat# 130-113-719)<br>CD45RA-BV786 : BD Bioscience (clone: HI100; cat# 563870)<br>CCR7-BB700 : BD Bioscience (clone: 3D12; cat# 566437)<br>CD27-BV421 : BioLegend (clone: O323; cat# 302823)<br>TCRαβ-FITC : BioLegend (clone: IP26; cat# 306705)<br>TCRγδ-PE-Cy7 : BioLegend (clone: B1; 331221)<br>Live/Dead Aqua Cell Stain : ThermoFisher Scientific (cat# L34957)
Validation	All antibodies are commercially available .<br>p24 KC57-PE : Beckman Coulter. Validated by our group: ref# Pardons et al. Plos Pathogens 2019<br>p24 28B7-APC : MediMabs. Validated by our group: ref# Pardons et al. Plos Pathogens 2019<br>CD3-AF700 : BD Bioscience. Manufacturer technical datasheet : "This antibody is routinely tested by flow cytometric analysis. Other applications were tested at BD Biosciences Pharmingen during antibody development only or reported in the literature."<br>CD8-FITC : Miltenyi/MACS. Manufacturer extend validation: https://www.miltenyibiotec.com/FI-en/products/cd8-antibody-anti-human-bw135-80.html#fitc:30-tests-in-60-ul<br>CD45RA-BV786 : BD Bioscience. Reference on BD website : Koristka S, Cartellieri M, Theil A, et al. Retargeting of human regulatory T cells by single-chain bispecific antibodies.. J Immunol. 2012; 188(3):1551-8<br>CCR7-BB700 : BD Bioscience. refrence on BD website : Ritter U, Wiede F, Mielenz D, Kiafard Z, Zwirner J, Korner H. Analysis of the

CCR7 expression on murine bone marrow-derived and spleen dendritic cells.. J Leukoc Biol. 2004; 76(2):472-476.
CD27-BV421 : BioLegend. Technical datasheet on the Biolegend website : https://www.biolegend.com/en-us/products/brilliant-violet-421-anti-human-cd27-antibody-7276
TCRαβ-FITC : BioLegend. Technical datasheet on the Biolegend website : https://www.biolegend.com/de-at/products/fitc-anti-human-tcr-alpha-beta-antibody-772
TCRγδ-PE-Cy7 : BioLegend. Technical datasheet on the Biolegend website : https://www.biolegend.com/de-at/products/pe-cyanine7-anti-human-tcr-gamma-delta-antibody-9113
Live/Dead Aqua Cell Stain : ThermoFisher Scientific. User guide on ThermoFischer website : https://www.thermofisher.com/document-connect/document-connect.html?url=https%3A%2F%2Fassets.thermofisher.com%2FTFS-Assets%2FLSG%2Fmanuals%2Flive_dead_fixable_dead_cell_stains_man.pdf&title=VXNlciBHdWlkZTogTElWRS9ERUFEIEZpeGFibGUgRGVhZCBDZWxsIFN0YWluIEtpdHM=
We performed titrations to determine optimal antibody concentrations.

Human research participants

Policy information about studies involving human research participants

Population characteristics — Eight individuals living with HIV on successful antiretroviral therapy (undetectable viremia for at least two years) were enrolled in this study. Age ranged between 32 and 67 years. Among the eight participant, one was a woman.
The study participants' characteristics are described in Supplementary Table 1.

Recruitment — No participants were recruited for this specific study. We used banked PBMC samples from the "Biobank to study HIV reservoirs" at the CRCHUM (N. Chomont), which allows to use PBMCs collected by leukapheresis for HIV reservoir studies. Participants who contributed to the biobank were recruited in two clinical centres (Fort Pierce, FL and Montréal QC). No selection was applied except for the criterias mentionned above (living with HIV and undetectable viremia for at least two years).

Ethics oversight — All participants were adults and signed informed consent forms approved by the McGill University Health Centre, the Centre Hospitalier de l'Université de Montréal and the Martin Memorial Health Systems review boards.

Note that full information on the approval of the study protocol must also be provided in the manuscript.

Flow Cytometry

Plots

Confirm that:

- [x] The axis labels state the marker and fluorochrome used (e.g. CD4-FITC).
- [x] The axis scales are clearly visible. Include numbers along axes only for bottom left plot of group (a 'group' is an analysis of identical markers).
- [x] All plots are contour plots with outliers or pseudocolor plots.
- [x] A numerical value for number of cells or percentage (with statistics) is provided.

Methodology

Sample preparation — PBMCs were isolated by Ficoll density gradient centrifugation and were cryopreserved in liquid nitrogen. CD4+ T cells were isolated by negative magnetic selection using the EasySep Human CD4+ T Cell Enrichment Kit (StemCell Technology, 19052). Purity was typically >98%. 5-15x106 CD4+ T cells were resuspended at 2x106 cells/mL in RPMI + 10% Fetal Bovine Serum and antiretroviral drugs were added to the culture medium (200nM raltegravir, 200nM lamivudine). Samples were pre-incubated for 1h with 5µg/mL Brefeldin A (BFA, Sigma, B2651) before stimulation in order to prevent the upregulation of cell surface markers, and BFA was maintained in the culture until the end of the stimulation. Cells were then stimulated with 1µg/mL ionomycin (Sigma, I9657) and 162nM PMA (24h) (Sigma, P8139). After stimulation, cells were collected, resuspended in PBS and stained with the Aqua Live/Dead staining kit for 30min at 4°C. Cells were then stained with antibodies against extracellular molecules in PBS + 4% human serum (Atlanta Biologicals, 540110) for 30min at 4°C. After a 45min fixation/permeabilization step was performed with the FoxP3 Transcription Factor Staining Buffer Set (eBioscience, 00-5523-00) following the manufacturer's instructions, cells were then stained with anti-p24 KC57 and anti-p24 28B7 antibodies for an additional 45min at RT in the FoxP3 Buffer. Cells were then washed and resuspended in PBS for subsequent cell sorting.

Instrument — BD FACS ARIA III

Software — Data collection: BD FACSDiva software
Data analysis: FlowJo v10.5.3

Cell population abundance — The frequency of p24 double positive cells (KC57+, 28B7+) ranged from 0.7 to 1,208 cells/million cells.

Gating strategy — All the relevant gating strategies are described in Supplementary Fig. 1b (p24+ cells and memory subsets) and Supplementary Fig. 2d (p24+ cells ad TCR alpha/beta and gamma/delta). Of note, CD4+ T cells from an HIV-uninfected control sample were included in each experiment to set the threshold of positivity for p24+ cells (double positive).

- [x] Tick this box to confirm that a figure exemplifying the gating strategy is provided in the Supplementary Information.
